# Improving community care for patients discharged from hospital through zone-wide implementation of a seamless care transition policy

**DOI:** 10.1093/intqhc/mzab079

**Published:** 2021-05-08

**Authors:** Naveenjyote Boora, Shireen Surood, Jeff Coulombe, Surya Poudel, Vincent I O Agyapong

**Affiliations:** Department of Psychiatry, Faculty of Medicine and Dentistry, University of Alberta, 8440 112 St NW, Edmonton AB T6G 2B7, Canada; Alberta Health Services, Addiction and Mental Health, 9942 - 108 Street, Edmonton AB T5K 2J5, Canada; Alberta Health Services, Addiction and Mental Health, 9942 - 108 Street, Edmonton AB T5K 2J5, Canada; Alberta Health Services, Addiction and Mental Health, 9942 - 108 Street, Edmonton AB T5K 2J5, Canada; Department of Psychiatry, Faculty of Medicine and Dentistry, University of Alberta, 8440 112 St NW, Edmonton AB T6G 2B7, Canada; Alberta Health Services, Addiction and Mental Health, 9942 - 108 Street, Edmonton AB T5K 2J5, Canada

**Keywords:** care transition policy, addiction and mental health, inpatient, community

## Abstract

**Background:**

Several studies within the psychiatry literature have illustrated the importance of discharge planning and execution, as well as accessibility of outpatient follow-up post-discharge. We report the results of implementing a new seamless care transition policy to expedite post-discharge follow-up in the community Addiction and Mental Health (AMH) program in the Edmonton Zone, Alberta, Canada. The policy involved a distribution mechanism for assessment by a mental health therapist (MHT) within 7 days of discharge as well as a dedicated roster of community psychiatrists to accept newly discharged patients.

**Objective:**

Our aim was to assess the feasibility of this novel policy and to assess its effect on our outcome measures of wait time to first outpatient MHT assessment and re-admission rate to hospital.

**Methods:**

Our study involved a retrospective clinical audit with total sampling design and a comparison of data 1 year before (2015/2016 fiscal year) and 1 year after (2017/2018 fiscal year) the implementation of the seamless care policy within the Edmonton Zone. Extracted data were analyzed with simple descriptive statistics and presented as percentages, mean and median.

**Results:**

Overall, with the enactment of this policy, follow-up volumes ultimately increased, while wait times for initial assessment decreased on average for patients discharged from the hospital. In the 2015/2016 fiscal year, MHT completed 128 assessments of post-discharge patients who were new to the community AMH program compared to 298 completed new assessments for the 2017/2018 fiscal year. The corresponding wait times for the new MHT assessments were 12.7 days (median of 12 days) and 7.8 days (median of 6 days), respectively. Similarly, psychiatrists completed only 59 assessments of post-discharge patients who were new to AMH compared to 133 new psychiatric assessments for the 2017/2018 fiscal year. The corresponding wait times for the new psychiatric assessments were 15.3 days (median of 14 days) and 8.8 days (median of 7 days), respectively. We correspondingly found a slight decline in readmission rates after the implementation of our model in the subsequent fiscal year.

**Conclusion:**

We envision that this policy will set a precedent with regard to streamlining post-discharge follow-up care for admitted inpatients, ultimately improving mental health outcomes for patients.

## Introduction

The impact of mental illness on Canadians is truly astounding. According to the Canadian Mental Health Association in 2013, one in five people in Canada will experience a mental health issue or illness. By age 40, ∼50% of the population will have or will have had a mental illness. Approximately 8% of Canadian adults will experience the debilitating mental illness known as major depressive disorder, and ∼1% of Canadians will experience bipolar disorder [1]. There is no doubt that mental illness is a great burden on our economy, our society and our health-care system. The economic toll is staggering in its own right—∼$51 billion per year is directed toward mental healthcare in Canada, resulting from health-care costs, lost productivity for those who are affected and reductions in quality of life [2]. The massive impact of mental illness on the lives of Canadians necessitates adequate access to care, especially after discharge from the hospital for a psychiatric illness. It has been shown that better outpatient access leads to reduced readmissions as well as reduction in the utilization of inpatient resources [3, 4].

While greater outpatient access for psychiatric patient’s post-discharge is a key issue, wait times for access to outpatient care is another. Reducing barriers to outpatient follow-up after discharge has been shown to result in lower costs for the health-care system along with improved quality of life for patients with outpatient follow-up in place [5]. Furthermore, these patients have a lower risk of readmission over time compared to patients without outpatient follow-up arranged at the time of discharge [6]. Thus, our objective is to address the issue of post-discharge outpatient follow-up with the implementation of a new seamless care transition policy. The goal of this policy is 2-fold, first to lower readmission rates for previously admitted inpatients and second to reduce wait times for the first psychiatric assessment in this population. Our study hypothesis was therefore that the introduction of the policy would achieve these two goals.

## Methods

### Study setting

The Edmonton Zone is one of five health administrative zones in Alberta, Canada, providing healthcare for about 1.4 million residents of the City of Edmonton and sub-urban cities. Addiction and Mental Health (AMH) services within the Edmonton Zone include a comprehensive continuum of services ranging from intensive inpatient services to community-based outpatient care. Ensuring a seamless and efficient continuum of care for our patients has been a long-standing priority for the zone. In January 2017, the AMH seamless care working group and AMH senior leadership team implemented a strategic initiative to expedite access to outpatient services for patients being discharged from psychiatric units. The initiative included the establishment of key processes whereby patients not previously connected to AMH outpatient services would be provided a follow-up appointment as soon as possible following discharge. The target was for patients to be seen by an AMH outpatient physician or therapist within 7 days of discharge. Key elements of the policy included the following:

- Development of a referral pathway and a referral form to be used by inpatient teams when a patient requires follow-up care post-discharge.
- Development of a roster of community psychiatrists who would accept a discharged patient into their schedule without prior approval when a 60-min block of time was available.
- Development of distribution mechanisms for new patients to be connected to a community therapist within 7 days of discharge.

Prior to the implementation of this initiative, patients being discharged from the hospital did receive follow-up care in the outpatient setting; however, no consistent processes or pathways were in place and patients often would have to go through the same process to access AMH services as patients from the community. Follow-up could be with existing AMH clinicians who they had a relationship with prior to hospitalization or could be new to AMH services. These clinicians could either be a mental health therapist (MHT) or a psychiatrist. The MHT designation applies to mental health social workers, registered nurses, occupational therapists and psychologists. Some patients do not access AMH services following discharge either because they declined follow-up care or are reconnected with clinicians (e.g. general practitioners and privately employed AMH clinicians) who do not record contacts in the Alberta Health Services AMH data systems.

### Study design

Our study involved a retrospective clinical audit with total sampling design and a comparison of data 1 year before (2015/2016 fiscal year) and 1 year after (2017/2018 fiscal year) the implementation of the seamless care policy within the Edmonton Zone. The fiscal year begins on 1 April and ends on 31 March of the subsequent year.

### Data collection and analysis

Data were extracted from the Discharge Abstract Database and eCLINICIAN, which are the electronic medical records (EMRs) used in the Edmonton Zone AMH services and presented to the research team as aggregated data. Specifically, the data used were collected from the wait time to Next Completed Appointment Tableau dashboard, Adult Community wait time report and Inpatient Readmission Rate Tableau dashboard. Extracted data were analyzed with simple descriptive statistics and presented as percentages, mean and median. This approach was utilized to compare discharge data from both the 2015/2016 fiscal year and the subsequent 2017/2018 fiscal year after the implementation of this policy.

## Results

For the 2015/2016 fiscal years, the Edmonton Zone recorded 4221 psychiatric inpatient discharges compared to 4148 discharges in the 2017/2018 fiscal year. Overall, 43.54% (1838/4221) and 50.12% (2079/4148) of these patients accessed outpatient AMH services of some type within 30 days of discharge in the year before and after the implementation of the seamless care transition policy, respectively. The corresponding wait times to follow-up in the community were 10 days (median of 7 days) and 8 days (median of 6 days), respectively.

### Comparison of post-discharge appointments completed for patients new to AMH services

In the 2015/2016 fiscal year, MHT completed 128 assessments of post-discharge patients who were new to AMH community compared to 298 completed new assessments for the 2017/2018 fiscal year. The corresponding wait times for the new MHT assessments were 12.7 days (median of 12 days) and 7.8 days (median of 6 days), respectively. Similarly, psychiatrists completed only 59 assessments of post-discharge patients who were new to the AMH community compared to 133 new psychiatric assessments for the 2017/2018 fiscal year. The corresponding wait times for the new psychiatric assessments were 15.3 days (median of 14 days) and 8.8 days (median of 7 days), respectively.

### Potential impact of the new policy on access and wait times for new patients overall

For the 2015/2016 fiscal year, for the whole AMH adult community program, regardless of referral source, there were 7287 new assessments by MHT compared to 8639 new MHT assessments in 2017/2018, representing an 18.55% increase in new MHT assessments in the fiscal year following the introduction of the new care transition policy. The corresponding mean wait times from referral to the assessments were 28 and 38 days, respectively.

Similarly, for the 2015/2016 fiscal year, there were 3254 new assessments by psychiatrists compared to 4594 new psychiatrist assessments in 2017/2018, representing a 41.18% increase in new psychiatrist assessments in the fiscal year following the introduction of the new care transition policy.

### Potential impact of the new policy on inpatient readmission rate within 30 days

The readmission rate was calculated as (number of readmissions occurring within 30 days of discharge/number of eligible stays) × 100 based on the index (prior) stay. For the fiscal year, 2015/2016, the readmission rate was 5.03% compared to 4.71% for 2017/2018, representing a marginal reduction in the readmission rate of 0.32%.

## Discussion

### Statement of principal findings

Overall, our goal with this study was to explore the implementation of a new policy aimed at expediting outpatient assessments for previously admitted psychiatric inpatients in the Edmonton Zone for AMH. Ultimately, the results show that our objective of reducing wait times for psychiatric assessments was met, as wait times were halved following the implementation of this new policy. Additionally, the uptake of new psychiatric inpatients into the outpatient system was increased substantially, with a 133% increase in new assessments by MHTs and a 125% increase in new assessments completed by psychiatrists. Similarly, there was an 18.55% increase in new MHT assessments and a 41.18% increase in new psychiatrist assessments in the community mental health program overall in the fiscal year following the introduction of the new care transition policy.

A hypothesis made at the outset of this initiative was that more prompt care following discharge would decrease the likelihood of inpatient readmission. Despite there being fewer discharged patients in the 2017/2018 fiscal year than the 2016/2017 fiscal year, the readmission rate was reduced modestly, at 0.32% from the previous year, with the implementation of this new policy. One would expect that although this was a modest change in readmission, further implementation of this policy over a longer period of time may yield further reduction in readmission rates for previously discharged inpatients.

### Strengths and limitations

A potential risk inherent in the initiative to expedite access for discharging patients is that the capacity to serve patients who are not in the hospital could be sacrificed in favor of prioritizing discharged patients as seen in the increased MHT wait times in our study. It was not possible to ascertain from the data if the increase in wait times overall for new MHT assessments for community patients was due to either an increase in the volume of new patients assessed post-introduction of the new policy or due to the prioritization of patients discharged from the hospital.

One limitation of our study is that, given the novelty of our policy and data lag from the provincial health records, we were only able to look at its impact over 1 fiscal year. Although the results from the 2017/2018 fiscal year are encouraging, further study of this policy over a longer period of time is warranted to ascertain the trajectory of readmission rates and outpatient wait times following discharge. In addition, since the researchers were receiving only aggregated data, it was not possible to test statistically significant differences in the data presented.

Another limitation of our study is that the corresponding mean wait times from referral to psychiatrist assessments are not available as most patients are referred to psychiatrists only after the assessment by an MHT is completed. Therefore, these data were not collected within the EMRs that were analyzed for this study.

Finally, a further limitation of this study is that in its current form, the data collected as part of this study are now at least 4–5 years old. The community mental health service under study has since 2019 introduced new programs to further expand access to care and improve the quality of care offered to clients at the community level. The most significant of these new initiatives was the introduction in January 2019 of the Addiction and Mental Health Access 24/7 program, which is manned by mental health therapists and psychiatrists 24 h a day and 7 days in a week. Consequently, adding data from subsequent years to this study would not allow for the assessment of the specific impact of the introduction of the care transition policy. Consequently, we are confident that the lack of recent data does not undermine this proof-of-concept study. Instead, our study promotes the introduction of care transition policies in other jurisdictions struggling to address similar challenges with their community mental health programs.

### Interpretation within the context of the wider literature

Notwithstanding the limitations of our study, our findings demonstrate the importance of this research that with effective policy, constructive changes can be made to reduce wait times for access to outpatient mental healthcare. This would ultimately contribute to a reduction in readmission for those patients. This research supports the broader psychiatry literature, which has demonstrated that greater access to follow-up outpatient care leads to improved mental health outcomes [7]. Without such a policy, many patients with subpar discharge planning may not have been able to access outpatient follow-up, which is a predictor of future readmission [8]. Future directions for this work would be a longer-term follow-up of this care transition policy with regard to readmissions and wait times as we have discussed here.

#### Integration of a systems-based approach to the findings

It is imperative that one consider the broader, system-level ramifications of this policy on how discharge systems are handled by various health-care systems. Although there may be a perceived trade-off where the quality of discharge follow-up is sacrificed in favor of speed, this work suggests that this is a false dichotomy. Commentaries of safety theorists such as Erik Hollnagel and the Efficiency–Thoroughness Trade Off (ETTO) fallacy do not support the notion of sacrificing efficiency for safety or thoroughness [9]. ETTO is a helpful way to conceptualize this perceived trade-off, and we draw attention to this in the hope that this will demonstrate to future health policymakers that this seamless-care transition policy effectively demonstrates a system in which safety and efficiency can both be feasibly prioritized.

### Implications for policy, practice and research

It is our hope that this policy will serve as a model policy for other jurisdictions to increase their uptake of discharged psychiatric inpatients so that they may have greater continuity of care and ultimately improved quality of life and functioning in the community. As mentioned above, there are implications for research in this field to provide a longer-term follow-up of patient readmission rates and uptake into community mental health resources to elucidate whether this is maintained for a sustained period after the implementation of this policy. We expect that this will influence those who are working in community mental health as either psychiatrists or mental health therapists to evaluate their transition coordination system and to advocate for improved access to expedient outpatient mental health care.

## Conclusions

The implementation of the care transition policy within the Edmonton Zone AMH services produced a 3-fold effect: it increased the volume of new patients connected for follow-up care following an acute hospitalization, reduced the mean wait times for these patients to access both MHT and psychiatrist assessments and produced a slight decline in readmission rates to the hospital. No evidence exists at present to indicate that services to the general population of patients accessing outpatient services have been negatively impacted by this policy. Overall, the volume of new patients receiving assessments from both psychiatrists and MHTs increased after the introduction of the policy. However, the overall wait times increased as well. The increase in wait times overall could have several contributory factors—the most probable of which is the increase in the volume of patients assessed rather than being a side effect of the introduction of the new policy.

## Data Availability

The primary data associated with this study are available on reasonable request from the Decision Support Services of Alberta Health Services, Addiction and Mental Health Department, Edmonton Zone.
